# Supplementary material for: Early-onset autoimmune vitiligo associated with an enhancer variant haplotype that upregulates class II HLA expression
Source: Nat Commun. 2019 Jan 23;10:391. doi: 10.1038/s41467-019-08337-4 (PMC6344500; doi:10.1038/s41467-019-08337-4)
Supplement: Supplementary file 1 — Supplementary Information [file 41467_2019_8337_MOESM1_ESM.pdf]

SUPPLEMENTARY INFORMATION

Early-Onset Autoimmune Vitiligo Associated with an Enhancer Variant Haplotype that Upregulates Class II HLA Expression

Jin et al.

SUPPLEMENTARY FIGURES

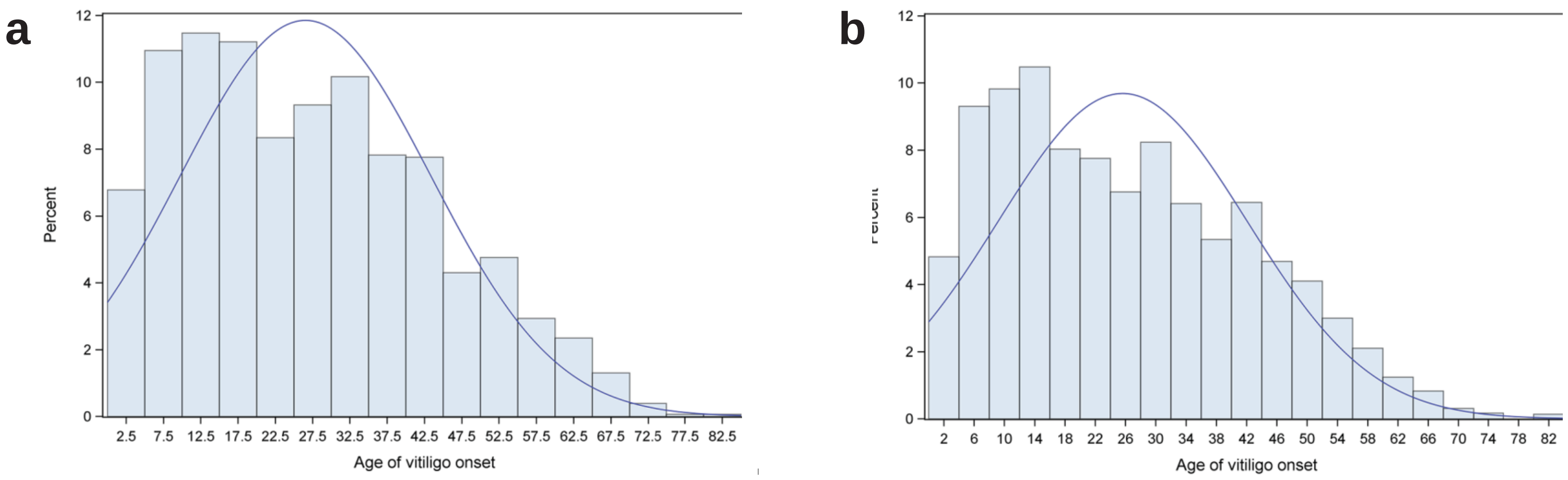

Supplementary Fig. 1 Distribution of vitiligo age-of-onset is bimodal in both males and females. The distribution of age-of-onset is shown for the total **a** 1,555 male cases (mean 26.5 years, SD 16.8) and **b** 2,968 female cases (mean 25.7 years, SD 16.5) from our three previous vitiligo GWAS and replication study[1-3]. Source data are provided as a Source Data file.

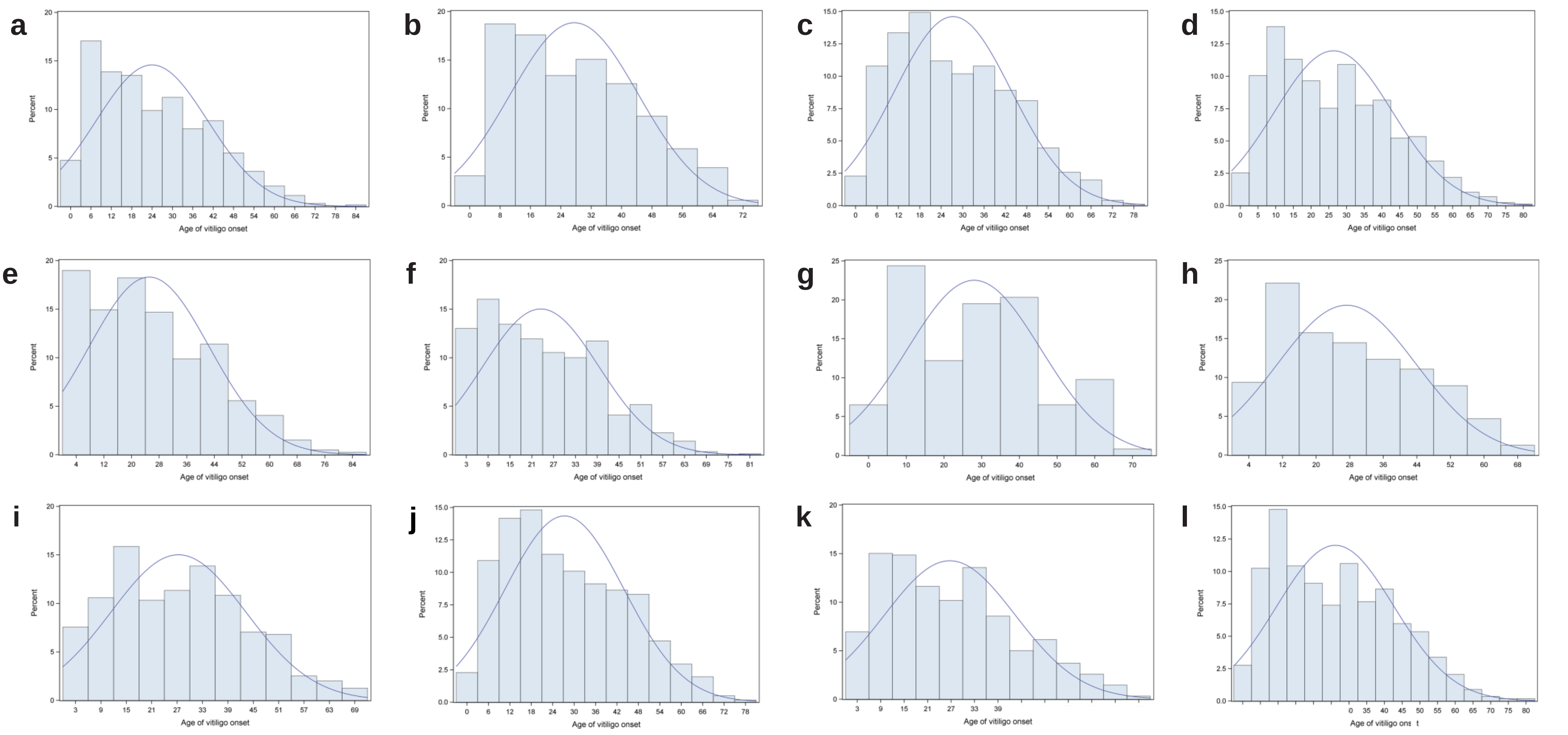

Supplementary Fig. 2 Distribution of vitiligo age-of-onset in the three previous GWAS and replication study case cohorts**.** The distributions of age-of-onset are shown for **a** 1,350 vitiligo cases (mean 24.1 years, SD 16.5) from our previous vitiligo GWAS1[1]. **b** 406 vitiligo cases (mean 27.7 years, SD 16.9) from our previous vitiligo GWAS2[2]. **c** 1,024 vitiligo cases (mean 27.2 years, SD 16.4) from our previous vitiligo GWAS3[3]. **d** 1,743 vitiligo cases (mean 26.3 years, SD 16.7) from our previous replication study[3]. **e** 399 male vitiligo cases (mean 25.1 years, SD 17.5) from our previous vitiligo GWAS1. **f** 951 female vitiligo cases (mean 23.6 years, SD 16.1) from our previous vitiligo GWAS1. **g** 138 male vitiligo cases (mean 28.0 years, SD 17.2) from our previous vitiligo GWAS2. **h** 268 female vitiligo cases (mean 27.5 years, SD 16.7) from our previous vitiligo GWAS2. **i** 397 male vitiligo cases (mean 27.3 years, SD 15.9) from our previous vitiligo GWAS3. **j** 627 female vitiligo cases (mean 27.2 years, SD 16.7) from our previous vitiligo GWAS3. **k** 621 male vitiligo cases (mean 26.6 years, SD 16.8) from our previous vitiligo GWAS3 replication study. **l** 1,122 female vitiligo cases (mean 26.1 years, SD 16.6) from our previous vitiligo GWAS3 replication study. Source data are provided as a Source Data file.

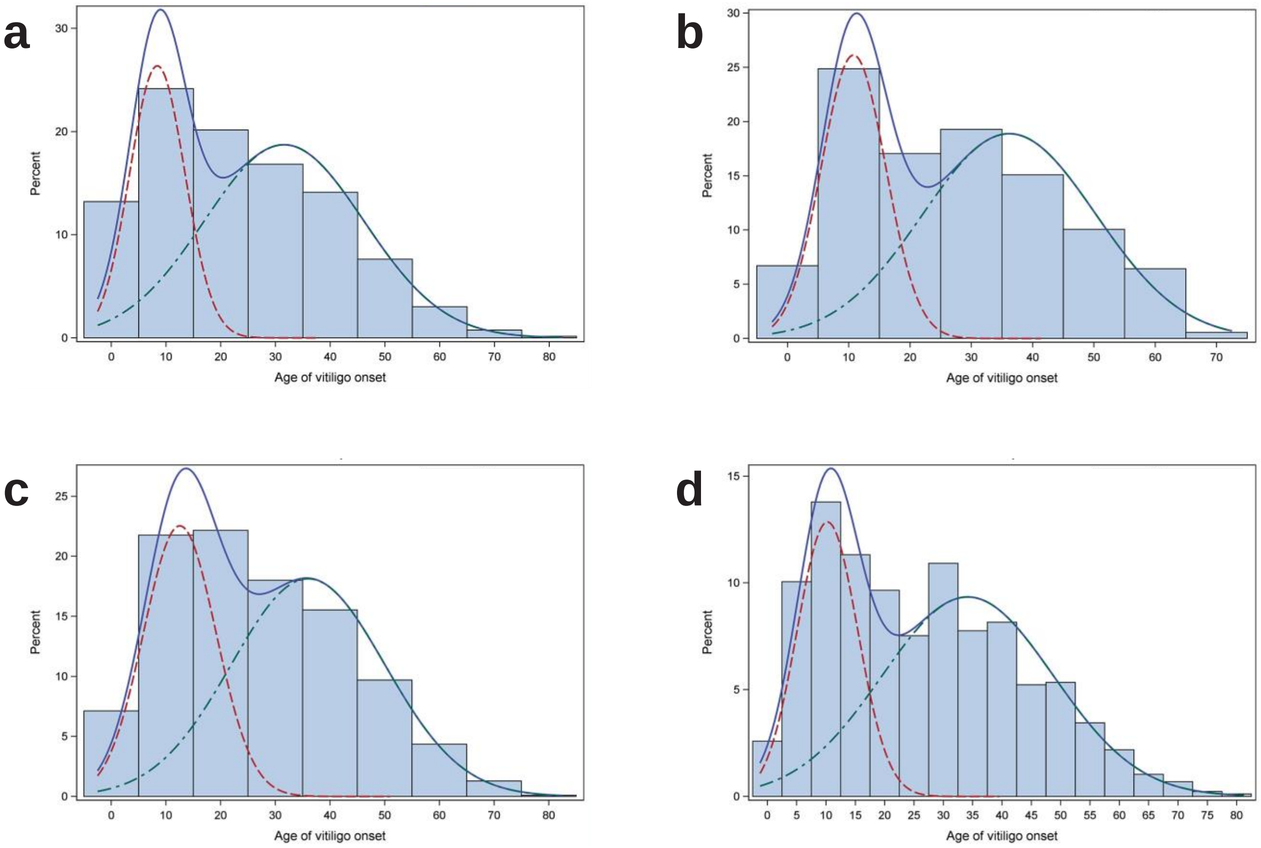

Supplementary Fig. 3 Distribution of vitiligo age-of-onset is bimodal in all four case cohorts. Best models from goodness of fit analysis in the three previous GWAS and replication study case cohorts[1-3]. Analyses were carried out by the finite mixture model (FMM) procedure in SAS. Blue lines show the full distributions, red lines the early-onset distributions, and green lines the late-onset distributions. **a** Best fit models for distribution of vitiligo age-of-onset in 1,350 vitiligo cases (mean 24.1 years, SD 16.5) from GWAS1. **b** Best fit models for distribution of vitiligo age-of-onset in 406 cases (mean 27.7 years, SD 16.9) from GWAS2. **c** Best fit models for distribution of vitiligo age-of-onset in 1,024 vitiligo cases (mean 27.2 years, SD years) from GWAS3. **d** Best fit models for distribution of vitiligo age-of-onset in 1,743 vitiligo cases (mean 26.3 years, SD 16.7) from GWAS3 replication study. Source data are provided as a Source Data file.

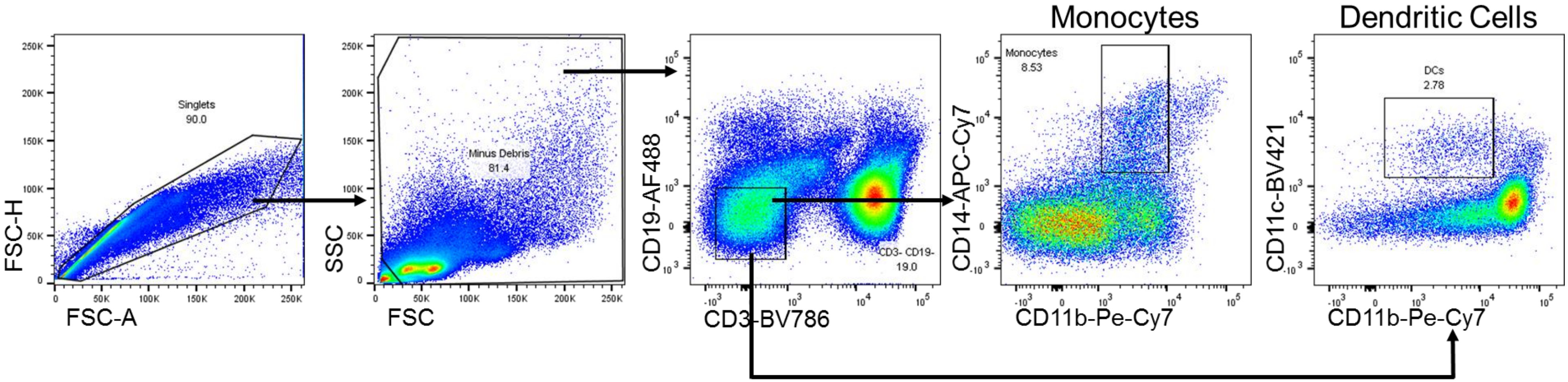

Supplementary Fig. 4 Gating strategies used for cell analysis. Forward scatter-area (FSC-A) by forward scatter-height (FSC-H) was used as a doublet discriminator. Mononuclear cells were selected based on size (FSC) and granularity (side scatter (SSC). To exclude T and B cells, the CD3 and CD19 double negative population was selected and either CD14 and CD11b double positive cells were used to identify monocytes ($CD3^-$, $CD11b^+$, $CD11c^-$, $CD14^+$, $CD19^-$) or CD11b negative CD11c positive cells were used to identify dendritic cells (DCs; $CD3^-$, $CD11b^-$, $CD11c^+$, $CD14^-$, $CD19^-$).

SUPPLEMENTARY TABLES

Supplementary Table 1 FMM component evaluation of mixture models supports bimodal distribution of vitiligo age-of-onset

Number of components	BIC				
	GWAS1	GWAS2	GWAS3	Replication study	Four cohorts combined
1	11415.64	3458.59	8645.19	14768.83	38278.87
2	11178.73	3399.85	8531.90	14469.60	37541.82
3	11200.35	3417.87	8552.70	14491.99	37567.07
4	11221.98	3435.88	8573.49	14514.38	37592.32
5	11243.60	3453.90	8594.29	14536.77	37617.57
6	11265.23	3471.92	8615.08	14559.16	37642.83
7	11286.85	3489.94	8635.88	14581.55	37668.08

FMM, Finite Mixture Model. BIC, Bayesian information criterion

Supplementary Table 2 Effect size differences at 49 previous confirmed vitiligo GWAS loci in the combined GWAS early-onset versus late-onset vitiligo subgroups

					Combined GWAS early-onset			Combined GWAS late-onset			$^{b}P_{Z}$ of OR difference
**Chr.**	**Variant**	**Position (Build 37)**	**Locus**	**EA/OA**	$^{a}P_{CMH}$	**OR**	**SE**	$^{a}P_{CMH}$	**OR**	**SE**	
1	rs301807	8484823	*RERE*	A/G	3.36 x 10^{-3}	1.19	0.06	2.30 x 10^{-5}	1.18	0.04	9.81 x 10^{-1}
1	rs2476601	114377568	*PTPN22*	A/G	2.39 x 10^{-3}	1.31	0.09	6.98 x 10^{-9}	1.41	0.06	5.04 x 10^{-1}
1	rs78037977	172715702	*FASLG*	G/A	4.49 x 10^{-7}	1.47	0.08	3.92 x 10^{-6}	1.29	0.05	1.54 x 10^{-1}
1	rs16843742	198672299	*PTPRC*	C/T	5.93 x 10^{-2}	0.88	0.07	2.07 x 10^{-3}	0.86	0.05	8.30 x 10^{-1}
2	rs10200159	55845109	*PPP4R3B*	C/T	1.48 x 10^{-4}	1.53	0.11	2.69 x 10^{-7}	1.47	0.08	7.70 x 10^{-1}
2	rs4308124	112010486	*BCL2L11*	C/T	5.72 x 10^{-7}	1.34	0.06	5.44 x 10^{-3}	1.12	0.04	1.11 x 10^{-2}
2	rs2111485	163110536	*IFIH1*	A/G	2.46 x 10^{-12}	0.65	0.06	1.56 x 10^{-9}	0.78	0.04	1.39 x 10^{-2}
2	rs231725	204740675	*CTLA4*	A/G	2.69 x 10^{-2}	1.14	0.06	3.44 x 10^{-5}	1.19	0.04	5.83 x 10^{-1}
2	rs41342147	242407588	*FARP2*	A/G	1.83 x 10^{-1}	0.89	0.09	9.81 x 10^{-4}	0.81	0.06	4.04 x 10^{-1}
3	rs35161626	23512312	*UBE2E2*	I/D	1.59 x 10^{-1}	0.92	0.06	3.85 x 10^{-6}	0.83	0.04	1.49 x 10^{-1}
3	rs34346645	71557945	*FOXP1*	A/C	2.67 x 10^{-2}	0.88	0.06	4.70 x 10^{-9}	0.79	0.04	1.27 x 10^{-1}
3	rs148136154	119283468	*CD80*	C/T	1.16 x 10^{-3}	1.31	0.08	1.65 x 10^{-6}	1.31	0.06	9.82 x 10^{-1}
3	rs13076312	188089254	*LPP*	T/C	2.95 x 10^{-6}	1.31	0.06	4.39 x 10^{-1}1	1.30	0.04	9.55 x 10^{-1}
3	rs6583331	196347253	*NRROS*	A/T	6.76 x 10^{-3}	0.85	0.06	2.40 x 10^{-4}	0.86	0.04	8.76 x 10^{-1}
4	rs1031034	102223386	*PPP3CA*	A/C	6.16 x 10^{-3}	0.83	0.07	9.39 x 10^{-4}	0.86	0.05	6.97 x 10^{-1}
6	rs12203592	396321	*IRF4*	T/C	6.62 x 10^{-2}	0.85	0.09	5.35 x 10^{-4}	0.81	0.06	6.68 x 10^{-1}
6	rs78521699	2908591	*SERPINB9*	G/A	2.50 x 10^{-1}	0.89	0.10	5.38 x 10^{-4}	0.78	0.07	2.74 x 10^{-1}
6	rs60131261	29937335	*HLA-A*	D/I	5.22 x 10^{-15}	1.58	0.06	6.94 x 10^{-23}	1.50	0.04	4.60 x 10^{-1}
6	rs9271597	32591291	*HLA-DRB1/DQA1*	A/T	1.95 x 10^{-37}	2.11	0.06	5.29 x 10^{-31}	1.58	0.04	6.00 x 10^{-5}
6	rs72928038	90976768	*BACH2*	A/G	7.50 x 10^{-2}	1.14	0.08	1.51 x 10^{-5}	1.24	0.05	3.64 x 10^{-1}
6	rs2247314	167370230	*RNASET2-FGFR1OP-CCR6*	C/T	8.31 x 10^{-9}	0.69	0.07	4.78 x 10^{-5}	0.84	0.04	1.16 x 10^{-2}
7	rs117744081	29132279	*CPVL*	G/A	1.34 x 10^{-3}	1.59	0.15	1.42 x 10^{-9}	1.83	0.10	4.36 x 10^{-1}
8	rs10087240	129012574	*PVT1*	T/C	5.99 x 10^{-1}	1.03	0.06	6.10 x 10^{-8}	1.24	0.04	9.02 x 10^{-3}
8	rs2687812	133931055	*SLA*	A/T	4.73 x 10^{-2}	1.12	0.06	1.45 x 10^{-9}	1.27	0.04	7.11 x 10^{-2}
9	rs10986311	127071493	*NEK6*	C/T	2.33 x 10^{-3}	1.20	0.06	9.29 x 10^{-3}	1.11	0.04	3.05 x 10^{-1}
10	rs706779	6098824	*IL2RA*	C/T	3.44x 10^{-9}	0.71	0.06	8.08 x 10^{-11}	0.77	0.04	2.17 x 10^{-1}
10	rs71508903	63779871	*ARID5B*	T/C	4.81x 10^{-1}	1.05	0.07	2.92 x 10^{-6}	1.25	0.05	4.72 x 10^{-2}
10	rs12771452	115488331	*CASP7*	A/G	5.42 x 10^{-3}	0.82	0.07	7.31 x 10^{-4}	0.85	0.05	6.79 x 10^{-1}
11	rs1043101	35274829	*CD44-SLC1A2*	G/A	5.72 x 10^{-2}	1.12	0.06	1.56 x 10^{-8}	1.25	0.04	1.09 x 10^{-1}

11	rs12421615	64021605	*PLCB3-BAD-GPR137*	A/G	6.92 x 10^{-2}	0.89	0.06	5.11 x 10^{-3}	0.89	0.04	9.52 x 10^{-1}
11	rs1126809	89017961	*TYR*	A/G	1.41 x 10^{-8}	0.67	0.07	8.47 x 10^{-17}	0.67	0.05	9.94 x 10^{-1}
11	rs11021232	95320808	*Gene desert*	C/T	4.94 x 10^{-6}	1.36	0.07	7.15 x 10^{-12}	1.38	0.05	8.61 x 10^{-1}
12	rs2017445	56407072	*IKZF4*	A/G	7.16 x 10^{-5}	1.27	0.06	4.11 x 10^{-9}	1.27	0.04	9.74 x 10^{-1}
12	rs10774624	111833788	*SH2B3*	A/G	1.92 x 10^{-4}	0.81	0.06	5.52x 10^{-9}	0.79	0.04	7.73 x 10^{-1}
13	rs35860234	43070206	*TTBK2*	G/T	1.93 x 10^{-2}	1.16	0.06	6.89 x 10^{-4}	1.16	0.04	1.00 x 10^{0}
14	rs8192917	25102160	*GZMB*	C/T	2.20 x 10^{-4}	1.27	0.07	2.56 x 10^{-7}	1.26	0.04	8.97 x 10^{-1}
15	rs1635168	28535266	*OCA2-HERC2*	A/C	5.76 x 10^{-4}	1.42	0.10	9.72 x 10^{-7}	1.41	0.07	9.59 x 10^{-1}
16	rs4268748	90026512	*MC1R*	C/T	5.29 x 10^{-12}	0.61	0.07	2.71 x 10^{-7}	0.79	0.05	3.31 x 10^{-3}
17	rs11079035	40289012	*RAB5C*	A/G	2.79 x 10^{-2}	1.18	0.07	6.11 x 10^{-3}	1.15	0.05	8.02 x 10^{-1}
18	rs8083511	60028655	*TNFRSF11A*	C/A	3.12 x 10^{-3}	1.23	0.07	2.12 x 10^{-5}	1.23	0.05	9.47 x 10^{-1}
19	rs4807000	4831878	*TICAM1*	A/G	5.16 x 10^{-2}	1.12	0.06	1.13 x 10^{-4}	1.17	0.04	5.71 x 10^{-1}
19	rs2304206	50168871	*IRF3-BCL2L12*	A/G	5.54 x 10^{-3}	0.82	0.07	6.56 x 10^{-5}	0.83	0.05	9.50 x 10^{-1}
20	rs6059655	32665748	*RALY-ASIP*	A/G	3.26 x 10^{-5}	0.59	0.13	7.65 x 10^{-6}	0.68	0.09	3.60 x 10^{-1}
20	rs6012953	49123043	*PTPN1*	G/A	6.41 x 10^{-2}	1.11	0.06	2.42 x 10^{-5}	1.18	0.04	3.85 x 10^{-1}
21	rs12482904	43851828	*UBASH3A*	A/T	9.00 x 10^{-5}	1.29	0.07	1.24 x 10^{-1}9	1.49	0.04	7.01 x 10^{-2}
22	rs229527	37581485	*C1QTNF6*	A/C	2.15 x 10^{-8}	1.38	0.06	1.73 x 10^{-1}2	1.32	0.04	5.54 x 10^{-1}
22	rs9611565	41767486	*ZC3H7B-TEF*	C/T	1.72 x 10^{-2}	0.83	0.07	2.65 x 10^{-10}	0.73	0.05	1.21 x 10^{-1}
X	rs73456411	29737404	*IL1RAPL1*	T/G	2.02 x 10^{-2}	1.57	0.20	1.71 x 10^{-5}	1.85	0.15	5.21 x 10^{-1}
X	rs5952553	49392721	*CCDC22-FOXP3*	C/T	5.85 x 10^{-3}	0.85	0.06	2.08 x 10^{-6}	0.82	0.04	6.83 x 10^{-1}

Chr., chromosome; EA, effect allele; OA, other allele; SE, standard error of the logarithm of the OR. The chromosome 16 association peak spans a large number of genes, including *MC1R*.

[a]P_{CMH}, Cochran-Mantel-Haenszel *P*-value

[b]P_Z, based on the statistic testing effect size (lnOR) difference between the earlier-onset and later-onset subgroups. See Methods for details.

Supplementary Table 3 Conditional effect size differences at the two principal MHC class II region variants in the combined GWAS and replication study early-onset and late-onset subgroups

				[a]Early-onset			[a]Late-onset			[b]Effect size difference	
**Chr.**	**Variant**	**Position (Build 37)**	**EA/OA**	$P_{conditional}$	**OR**	**SE**	$P_{conditional}$	**OR**	**SE**	Z **score**	P_Z
Combined GWAS											
6	rs145954018	32440321	D/I	1.01×10^{-31}	3.35	0.10	0.16	1.15	0.10	7.61	2.77×10^{-14}
6	rs9271597	32591291	A/T	2.99×10^{-24}	1.91	0.06	2.04×10^{-31}	1.62	0.04	2.19	0.03
Replication Study											
6	rs145954018	32440321	D/I	1.57×10^{-19}	4.10	0.16	1.29×10^{-1}	1.30	0.17	4.91	9.11×10^{-7}
6	rs9271597	32591291	A/T	6.42×10^{-12}	1.93	0.10	1.60×10^{-8}	1.53	0.07	1.93	0.05

Chr., chromosome; EA, effect allele; OA, other allele; SE, standard error of the logarithm of the OR.

[a]Obtained with both variants and significant eigenvectors from each genomewide association study in the same logistic regression model for the combined GWAS, and both variants in the same model for the replication study.

[b]A Z test[4] was used to test effect size difference, with Z calculated as the difference between the logarithms of the ORs, divided by the square root of the sum of the variances of the logarithms of the ORs, where Z follows a normal distribution.

Supplementary Table 4 Comparing additive and dominant effect models for vitiligo risk for the rs145954018del-rs9271597A haplotype

**Additive effect model**			
	[a]$A_{rs145954018del\text{-}rs9271597A}$		
OR (95% CI)	7.24 (6.02-8.71)		
$P_{logistic}$	1.70 x 10^{-85}		
[d]BIC			6125.18
**Additive effect model with dominance term**			
	[a]$A_{rs145954018del\text{-}rs9271597A}$	[b]$D_{rs145954018del\text{-}rs9271597A}$	
OR (95% CI)	4.10 (2.61-6.44)	1.92 (1.18-3.12)	
$P_{logistic}$	1.70 x 10^{-85}	1.06 x 10^{-2}	
[d]BIC			6128.02
**Complete dominant effect model**			
	[c]$DD_{rs145954018del\text{-}rs9271597A}$		
OR (95% CI)	8.10 (6.71-9.79)		
$P_{logistic}$	2.40 x 10^{-86}		
[d]BIC			6121.27

Data were from the combined GWAS and replication study early-onset subgroups.
[a]A, coded as 0, 1, 2 for having 0, 1, or 2 copies
[b]D, coded as 0, 1, 0 for having 0, 1, or 2 copies
[c]DD, coded as 0, 1, 1 for having 0, 1, or 2 copies
[d]BIC, Bayesian information criterion.

Supplementary Table 5 Sequencing of classical MHC class II *HLA* alleles in 20 European-ancestry vitiligo cases selected based on rs9271597 genotypes

Case	Age of Onset	rs145954018	*DRB1*	*DRB3/4/5*	rs9271597	*DQA1*	*DQB1*	*DPB1*
1	25	del	DRB1*13:01	DRB3*01:01	A	DQA1*01:03	DQB1*06:03	DPB1*04:01
		del	DRB1*13:01	DRB3*02:02	A	DQA1*01:03	DQB1*06:03	DPB1*04:01
2	7	del	DRB1*13:01	DRB3*01:01	A	DQA1*01:03	DQB1*06:03	DPB1*04:01
		TG	DRB1*04:01	DRB4*01:01	A	DQA1*03:01	DQB1*03:01	DPB1*02:01
3	13	del	DRB1*13:01	DRB3*01:01	A	DQA1*01:03	DQB1*06:03	DPB1*04:01
		TG	DRB1*04:01	DRB4*01:01	A	DQA1*03:01	DQB1*03:01	DPB1*04:01
4	11	del	DRB1*13:01	DRB3*01:01	A	DQA1*01:03	DQB1*06:03	DPB1*04:01
		TG	DRB1*04:01	DRB4*01:01	A	DQA1*03:01	DQB1*03:01	DPB1*14:01
5	4	del	DRB1*13:01	DRB3*01:01	A	DQA1*01:03	DQB1*06:03	DPB1*04:01
		TG	DRB1*04:03	DRB4*01:01	A	DQA1*03:01	DQB1*03:04	DPB1*02:01
6	4	del	DRB1*13:01	DRB3*01:01	A	DQA1*01:03	DQB1*06:03	DPB1*02:01
		TG	DRB1*07:01	DRB4*01:01	A	DQA1*02:01	DQB1*02:02	DPB1*14:01
7	5	del	DRB1*13:01	DRB3*01:01	A	DQA1*01:03	DQB1*06:03	DPB1*11:01
		TG	DRB1*07:01	DRB4*01:01	A	DQA1*02:01	DQB1*02:02	DPB1*10:01
8	4	del	DRB1*13:01	DRB3*01:01	A	DQA1*01:03	DQB1*06:03	DPB1*11:01
		TG	DRB1*07:01	DRB4*01:01	A	DQA1*02:01	DQB1*02:02	DPB1*20:01
9	17	TG	DRB1*04:01	DRB4*01:01	A	DQA1*03:01	DQB1*03:01	DPB1*02:01
		TG	DRB1*04:01	DRB4*01:01	A	DQA1*03:01	DQB1*03:01	DPB1*04:01
10	16	TG	DRB1*04:01	DRB4*01:01	A	DQA1*03:01	DQB1*03:01	DPB1*01:01
		TG	DRB1*04:01	DRB4*01:01	A	DQA1*03:01	DQB1*03:02	DPB1*04:01
11	11	TG	DRB1*04:01	DRB4*01:01	A	DQA1*03:01	DQB1*03:01	DPB1*04:01
		TG	DRB1*04:08	DRB4*01:01	A	DQA1*03:01	DQB1*03:01	DPB1*04:01
12	34	TG	DRB1*04:01	DRB4*01:01	A	DQA1*03:01	DQB1*03:01	DPB1*04:01
		TG	DRB1*04:04	DRB4*01:01	A	DQA1*03:01	DQB1*03:02	DPB1*06:01
13	40	TG	DRB1*04:01	DRB4*01:01	A	DQA1*03:01	DQB1*03:01	DPB1*04:02
		TG	DRB1*07:01	DRB4*01:01	A	DQA1*02:01	DQB1*02:02	DPB1*11:01
14	36	TG	DRB1*04:01	DRB4*01:01	A	DQA1*03:01	DQB1*03:01	DPB1*04:01
		TG	DRB1*07:01	DRB4*01:01	A	DQA1*02:01	DQB1*03:03	DPB1*04:02
15	28	TG	DRB1*07:01	DRB4*01:01	A	DQA1*02:01	DQB1*02:02	DPB1*11:01
		TG	DRB1*07:01	DRB4*01:01	A	DQA1*02:01	DQB1*02:02	DPB1*16:01
16	4	TG	DRB1*07:01	DRB4*01:01	A	DQA1*02:01	DQB1*02:02	DPB1*04:01
		TG	DRB1*07:01	DRB4*01:01	A	DQA1*02:01	DQB1*03:03	DPB1*04:01
17	30	TG	DRB1*07:01	DRB4*01:01	A	DQA1*02:01	DQB1*03:03	DPB1*04:01
		TG	DRB1*13:02	DRB3*03:01	A	DQA1*01:02	DQB1*06:04	DPB1*02:01
18	22	TG	DRB1*07:01	DRB4*01:01	A	DQA1*02:01	DQB1*03:03	DPB1*03:01
		TG	DRB1*13:02	DRB3*03:01	A	DQA1*01:02	DQB1*06:04	DPB1*04:01
19	55	TG	DRB1*13:02	DRB3*03:01	A	DQA1*01:02	DQB1*06:04	DPB1*03:01
		TG	DRB1*04:02	DRB4*01:01	A	DQA1*03:01	DQB1*03:02	DPB1*05:01
20	13	TG	DRB1*07:01	DRB4*01:01	A	DQA1*02:01	DQB1*02:01	DPB1*13:01
		TG	DRB1*13:03	DRB3*01:01	T	DQA1*05:01	DQB1*03:01	DPB1*15:01

Mean age-of-onset in subjects carrying the early-onset associated high-risk rs145954018del-rs9271597A haplotype (n = 8) was 9.1 years, SD 2.6, and in subjects not carrying the high-risk haplotype (n = 12) was 25.4 years, SD 14.2. Blue, early-onset associated high-risk rs145954018del—*HLA-DRB1*13:01*—*HLA-DRB3*01:01*—rs9271597A—*HLA-DQA1*01:03*—*HLA-DQB1*06:03* haplotype; green, yellow, and orange, late-onset associated rs145954018TG-rs9271597A high-risk haplotypes. Sequencing of MHC class II *HLA* classical alleles was described previously[5]. Haplotypes are based on international and European-American high-resolution extended *HLA* haplotype standards[6].
Note: These cases are not necessarily the same as those studied by RNA-seq and protein expression analyses.

Supplementary Table 6 Comparing fit of haplotype models defined by different combinations of variants in the combined GWAS early-onset subset

Variants defining haplotypes	Haplotype	Frequency	[a]OR (95% CI)	[b]$P_{logistic}$	BIC
rs145954018, rs9271597	DA	0.039	7.80 (6.34-9.60)	1.36×10^{-53}	
	IA	0.392	2.01 (1.77-2.28)	7.95×10^{-12}	
	IT	0.568	reference		4385.35
*DRB1*13:01*, *DQA1*01:03*, *DQB1*06:03*	PPP	0.068	3.20 (2.71-3.77)	1.18×10^{-43}	
	AAA	0.922	reference		4585.84
rs145954018, *DRB1*13:01*, rs9271597	DPA	0.039	7.66 (6.23-9.44)	3.81×10^{-53}	
	IPA	0.030	3.17 (2.42-4.14)	4.45×10^{-3}	
	IAA	0.362	1.93 (1.69-2.19)	3.48×10^{-9}	
	IAT	0.568	reference		4389.26
rs145954018, rs9271597, *DQA1*01:03*	DAP	0.039	7.42 (6.09-9.15)	3.46×10^{-53}	
	IAP	0.030	3.51 (2.72-4.53)	4.52×10^{-3}	
	IAA	0.362	1.88 (1.64-2.14)	4.99×10^{-9}	
	ITA	0.561	reference		4395.85
rs145954018, rs9271597, *DQB1*06:03*	DAP	0.038	7.16 (5.81-8.83)	2.11×10^{-53}	
	IAP	0.031	3.98 (3.10-5.09)	5.01×10^{-3}	
	IAA	0.361	1.82 (1.60-2.08)	4.44×10^{-9}	
	ITA	0.566	reference		4396.98
rs145954018, *DRB1*13:01*, rs9271597, *DQA1*01:03*, *DQB1*06:03*	DPAPP	0.038	6.96 (5.65-8.58)	1.97×10^{-53}	
	IPAPP	0.030	3.03 (2.32-3.96)	3.82×10^{-3}	
	IAAAA	0.361	1.82 (1.60-2.07)	3.19×10^{-9}	
	IATAA	0.560	reference		4430.90

Allele codes in haplotypes: for *HLA-DRB1*13:01*, *-DQA1*01:03*, and *-DQB1*06:03*, P = Present or A = Absent; for rs145954018, D = del or I = TG; for rs9271597, A or T. BIC, Bayesian information criterion.
[a]Obtained by comparing with the reference haplotype in the model.
[b]Obtained by testing this haplotype against all other haplotypes as one group in the model.

Supplementary Table 7 Frequencies of other vitiligo-associated autoimmune diseases in vitiligo cases in the early-onset and late-onset subgroups

	[a]Early-onset vitiligo cases (n = 914)		[a]Late-onset vitiligo cases (n = 2,966)		
**Phenotype**	**n**	**%**	**n**	**%**	[c]P_{Fisher}
Vitiligo with type 1 diabetes	19	2.1	64	2.2	0.80
Vitiligo with rheumatoid arthritis	13	1.4	62	2.1	0.17
Vitiligo with systemic lupus erythematosus	2	0.2	8	0.3	1.00
Vitiligo with Addison's disease	5	0.5	16	0.5	1.00
Vitiligo with pernicious anemia	14	1.5	53	1.8	0.57
Vitiligo with autoimmune thyroid disease[b]	124	13.6	543	18.3	6.27×10^{-4}
Vitiligo with at least one of the six other autoimmune diseases	155	17.0	665	22.4	4.05×10^{-4}

[a]Cases from GWAS1 GWAS2, GWAS3, and the replication study for whom data regarding presence or absence of other autoimmune diseases were available.

[b]Cases recorded as having Graves' disease, Hashimoto's thyroiditis, or hypothyroidism, most of which would be Hashimoto's thyroiditis. The mean ages of vitiligo onset were very similar in these case groups.

[c]*P*-value from Fisher's exact test comparing early-onset and late-onset subgroups.

SUPPLEMENTARY REFERENCES

1. Jin, Y., et al. Variant of *TYR* and autoimmunity susceptibility loci in generalized vitiligo. *N. Engl. J. Med*. **362**, 1686-1697 (2010).
2. Jin, Y., et al. Genome-wide association analyses identify 13 new susceptibility loci for vitiligo. *Nat. Genet*. **44**, 676–680 (2012).
3. Jin, Y., et al. Genome-wide association studies of autoimmune vitiligo identify 23 new risk generalized loci and highlight key pathways and regulatory variants. *Nat. Genet*. **48**, 1418-1424 (2016).
4. Paternoster, R., Brame, R., Mazerolle, P., & Piquero, A. Using the correct statistical test for the equality of regression coefficients. *Criminology* **36**, 859-866 (1998).
5. Cavalli, G., et al. MHC class II super-enhancer increases surface expression of HLA-DR and HLA-DQ and affects cytokine production in autoimmune vitiligo. *Proc. Natl. Acad. Sci. U.S.A.* **113**, 1363-1368 (2016).
6. Klitz, W., et al. New HLA haplotype frequency reference standards: High-resolution and large sample typing of HLA DR-DQ haplotypes in a sample of European Americans. *Tissue Antigens* **62**, 296-307 (2003).
