# Supplementary material for: Early-onset autoimmune vitiligo associated with an enhancer variant haplotype that upregulates class II HLA expression
Source: Nat Commun. 2019 Jan 23;10:391. doi: 10.1038/s41467-019-08337-4 (PMC6344500; doi:10.1038/s41467-019-08337-4)
Supplement: Supplementary file 5 — Description of Additional Supplementary Files [file 41467_2019_8337_MOESM5_ESM.pdf]

Description of Additional Supplementary Files

File Name: Supplementary Data 1
Description: Chr., chromosome; EA, effect allele; OA, other allele; MAF, minor allele frequency; SE, standard error of the logarithm of the OR. For classical HLA alleles and amino acids, P = Present, A = Absent. PCMH, Cochran-Mantel-Haenszel P-value. *A Z test was used to test effect size difference, with Z calculated as the difference between the logarithms of the ORs, divided by the square root of the sum of the variances of the logarithms of the ORs, where Z follows a normal distribution.

File Name: Supplementary Data 2
Description: All variants were tested that achieved genomewide significant association in either or both the early-onset and late-onset subgroups. Chr., chromosome; EA, effect allele; OA, other allele; SE, standard error of the logarithm of the OR; NA, not available. For HLA classical alleles and amino acids, P = Present, A = Absent. Genomewide significance was defined as PCMH < 5.0E-08. a Obtained with SNPs rs145954018 and rs9271597 as covariates in the model. b A Z test was used to test effect size difference, with Z calculated as the difference between the logarithms of the ORs, divided by the square root of the sum of the variances of the logarithms of the ORs, where Z follows a normal distribution.
