# Supplementary material for: Early-onset autoimmune vitiligo associated with an enhancer variant haplotype that upregulates class II HLA expression
Source: Nat Commun. 2019 Jan 23;10:391. doi: 10.1038/s41467-019-08337-4 (PMC6344500; doi:10.1038/s41467-019-08337-4)
Supplement: Supplementary file 6 — Reporting Summary [file 41467_2019_8337_MOESM6_ESM.pdf]

nature research

Corresponding author(s): Richard A. Spritz

Reporting Summary

Nature Research wishes to improve the reproducibility of the work that we publish. This form provides structure for consistency and transparency in reporting. For further information on Nature Research policies, see Authors & Referees and the Editorial Policy Checklist.

Statistical parameters

When statistical analyses are reported, confirm that the following items are present in the relevant location (e.g. figure legend, table legend, main text, or Methods section).

n/a	Confirmed	
☐	☒	The exact sample size (n) for each experimental group/condition, given as a discrete number and unit of measurement
☐	☒	An indication of whether measurements were taken from distinct samples or whether the same sample was measured repeatedly
☐	☒	The statistical test(s) used AND whether they are one- or two-sided<br>*Only common tests should be described solely by name; describe more complex techniques in the Methods section.*
☐	☒	A description of all covariates tested
☐	☒	A description of any assumptions or corrections, such as tests of normality and adjustment for multiple comparisons
☐	☒	A full description of the statistics including central tendency (e.g. means) or other basic estimates (e.g. regression coefficient) AND variation (e.g. standard deviation) or associated estimates of uncertainty (e.g. confidence intervals)
☐	☒	For null hypothesis testing, the test statistic (e.g. F, t, r) with confidence intervals, effect sizes, degrees of freedom and P value noted<br>*Give P values as exact values whenever suitable.*
☒	☐	For Bayesian analysis, information on the choice of priors and Markov chain Monte Carlo settings
☒	☐	For hierarchical and complex designs, identification of the appropriate level for tests and full reporting of outcomes
☐	☒	Estimates of effect sizes (e.g. Cohen's d, Pearson's r), indicating how they were calculated
☐	☒	Clearly defined error bars<br>*State explicitly what error bars represent (e.g. SD, SE, CI)*

Our web collection on statistics for biologists may be useful.

Software and code

Policy information about availability of computer code

Data collection	GeneMapper®, version 4.0 Real-Time Analysis software (version 3.3.3) (Applied Biosystems); bcl2fastq2 Conversion Software, v2.20.0 https://support.illumina.com/downloads/bcl2fastq-conversion-software-v2-20.html
Data analysis	SAS, version 9.4; R (v3.3.3) https://www.r-project.org; Stata, version 10.1 (https://www.stata.com/); PLINK (Purcell et al., 2007), version 1.9; EIGENSOFT (Price et al., 2006); IMPUTE2 (Howie et al., 2011); Eagle2 (Loh et al., 2016); PBWT (Durbin, 2014); SNP2HLA (Jia et al., 2013); GemTools (Klei et al., 2011); R, version 3.1.3; GCTA (Yang et al., 2011); seq2HLA, version 2.2 (Boegel et al., 2012); Sanger Imputation Service https://imputation.sanger.ac.uk/; Haplotype Reference Consortium reference panel (release 1.1) http://www.haplotype-reference-consortium.org/; 1000 Genomes Project phase I integrated variant set (version 3, March, 2012) http://www.1000genomes.org; International HapMap Project http://hapmap.ncbi.nlm.nih.gov/; NHGRI-EBI Catalog of published genome-wide association studies https://www.ebi.ac.uk/gwas/downloads/summary-statistics; dplyr (R package; v0.7.4) https://dplyr.tidyverse.org; ggplot2 (R package; v2.2.1) https://ggplot2.tidyverse.org/authors.html; ggbio (R package; v1.22.4) https://bioconductor.org/packages/release/bioc/html/ggbio.html; GenomicRanges (R package; v1.26.4) https://bioconductor.org/packages/release/bioc/html/GenomicRanges.html; biovizBase (R package; v1.22.0) https://bioconductor.org/packages/release/bioc/html/biovizBase.html; Homo.Sapiens (R package; v1.3.1) https://bioconductor.org/packages/release/data/annotation/html/Homo.sapiens.html; Homo.Sapiens (R package; v1.3.1) https://bioconductor.org/packages/release/data/annotation/html/Homo.sapiens.html

For manuscripts utilizing custom algorithms or software that are central to the research but not yet described in published literature, software must be made available to editors/reviewers upon request. We strongly encourage code deposition in a community repository (e.g. GitHub). See the Nature Research guidelines for submitting code & software for further information.

Data

Policy information about availability of data

All manuscripts must include a data availability statement. This statement should provide the following information, where applicable:

- Accession codes, unique identifiers, or web links for publicly available datasets
- A list of figures that have associated raw data
- A description of any restrictions on data availability

Data Availability
Case genotype and phenotype data for GWAS1, GWAS2, and GWAS3 subjects, and RNA-seq data for the present study, have been deposited in the Database of Genotypes and Phenotypes (dbGaP) under accession numbers phs000224.v1.p1, phs000224.v2.p1, phs000224.v3.p2 , and phs000224.v4.p2 [https://www.ncbi.nlm.nih.gov/projects/gap/cgi-bin/study.cgi?study_id=phs000224.v3.p2]). Vitiligo susceptibility and age-of-onset GWAS summary statistics have been deposited in the NHGRI-EBI Catalog of published genome-wide association studies [https://www.ebi.ac.uk/ega/studies/phs000224.v1.p1].

Field-specific reporting

Please select the best fit for your research. If you are not sure, read the appropriate sections before making your selection.

☒ Life sciences ☐ Behavioural & social sciences ☐ Ecological, evolutionary & environmental sciences

For a reference copy of the document with all sections, see nature.com/authors/policies/ReportingSummary-flat.pdf

Life sciences study design

All studies must disclose on these points even when the disclosure is negative.

Sample size	Samples for genetic analyses included all subjects from previous GWAS1 (1,381 cases), GWAS2 (413 cases), GWAS3 (1,059 cases), and replication study (1,743 cases and 2,182 unaffected controls). Samples for protein analyses included 46 available control subjects from the replication study who carried relevant rs145954018 and rs9271601 genotypes and haplotypes.
Data exclusions	Quality control filtering of genome-wide genotype data was carried out using PLINK, version 1.9. For each case/control dataset, DNA strand calls were reversed as needed. Cases were excluded on the basis of SNP call rates < 98.5%, discordance between reported and observed sex, or inadvertent subject duplication, and controls were excluded on the basis of SNP call rates < 98%. SNPs were excluded on the basis of genotype missing rate > 2% for SNPs with observed minor allele frequency (MAF) > 0.01, and for SNPs with MAF < 0.01 exclusion criteria were genotype missing rate >1% and < 5 minor alleles observed, or significant (P < E−4) deviation from Hardy-Weinberg equilibrium. For X chromosome SNPs, Hardy-Weinberg equilibrium tests were performed in females, and SNPs with P < E−4 were excluded from the final analysis. For each GWAS, only SNPs that existed in all case and control datasets were retained for imputation. Within each GWAS, subjects were excluded based on cryptic relatedness identified by pairwise identity-by-descent estimations (pi-hat > 0.0625), in which case the individual with lower SNP call rate was excluded. For each of the three GWAS, the cleaned case dataset was combined with one cleaned control dataset at a time and the genotype data of 270 subjects of Phase I and II of the International HapMap Project from 4 populations (http://hapmap.ncbi.nlm.nih.gov/), and principal components analysis (PCA) was performed with EIGENSOFT based on tag-SNPs (within which no pair were correlated with r2 > 0.2) selected from genotyped SNPs. The first two eigenvectors were used to produce a PCA plot. A PCA plot was first made for cases and HapMap samples, and cases that were clearly separated from the main cluster of cases and HapMap EUR samples were excluded as outliers. A PCA plot of controls and HapMap samples was then made, and the same x and y coordinates that separated the case outliers from the main cluster of cases and HapMap EUR samples were used to identify control outliers. After all QC procedures, the final number of genotyped SNPs remaining in GWAS1, GWAS2, and GWAS3 were 464,902, 494,043, and 483,609, respectively
Replication	In the replication study, rs145954018 and rs9271597 were genotyped using the Applied Biosystems SNaPshot Multiplex System. Both variants had genotype call rates > 95% and were in Hardy-Weinberg equilibrium (P > 0.05). We then performed logistic regression analyses of rs145954018 and rs9271597 genotypes in the early- and late-onset subgroups. The replication study yielded highly significant replication results.
Randomization	To compensate for sample size differences between the two subgroups, we used simple random sampling implemented in the SURVEYSELECT procedure in SAS version 9.4 to randomly assign two-thirds (n = 1,455) of controls to the early-onset subgroup and one-third (n = 727) to the late-onset subgroup.
Blinding	Analyses were not blind.

Reporting for specific materials, systems and methods

Materials & experimental systems

n/a	Involved in the study
☒	☐ Unique biological materials
☐	☒ Antibodies
☒	☐ Eukaryotic cell lines
☒	☐ Palaeontology
☒	☐ Animals and other organisms
☐	☒ Human research participants

Methods

n/a	Involved in the study
☒	☐ ChIP-seq
☐	☒ Flow cytometry
☒	☐ MRI-based neuroimaging

Antibodies

Antibodies used

PerCP-Cy5.5 anti-CD1c (BioLegend Cat# 331514, RRID AB_1227535); Brilliant Violet 785 anti-CD3 (BioLegend Cat# 317330; RRID AB_2563507); PE-Cy7 anti-CD11b (BioLegend Cat# 301322; RRID AB_830644); Brilliant Violet 421 anti-CD11c (BioLegend Cat# 301628; RRID AB_11203895); APC-Cy7 anti-CD14 (BioLegend Cat# 367108; RRID AB_2566710); Alexa Fluor 488 anti-CD19 (EBioscience Cat# 53-0199-42; RRID AB_1659677); APC anti-HLA-DR (BioLegend Cat# 307610; RRID AB_314688); PE anti-HLA-DQ (BioLegend Cat# 318106; RRID AB_604129). All antibodies were used undiluted.

Validation

We have verified these antibodies to measure expression of surface markers of peripheral blood mononuclear cells by flow cytometry analysis in a number of our previous publications (PMID: 29650491, 26787888, 20656923). Here, we used a verified staining protocol, and we additionally tested cells isolated from a single donor across all sample batches. These cells consistently stained within a narrow range for all surface markers across all batches. For example, percent of CD19 cells ranged from 3.82-4.06 and MFI of HLA-DR and HLA-DQ was within 20% across all sample time points.

Human research participants

Policy information about studies involving human research participants

Population characteristics

All subjects were unrelated and of self-described non-Hispanic/Latino European-derived white ancestry from North America and Europe. There were no age or sex restrictions. All cases met diagnostic criteria for generalized vitiligo; specifically, acquired multifocal patches of depigmented skin, typically bilateral but not necessarily symmetric, a waxing and waning but generally progressive clinical course, and with no known exposure to depigmenting chemicals. Subjects with apparent segmental vitiligo, and with piebaldism, Waardenburg syndrome, or other monogenic syndromes that include congenital patchy skin depigmentation were excluded. All vitiligo cases provided self-reported vitiligo age-of-onset.

Recruitment

All vitiligo cases and non-vitiligo controls were enrolled for previous GWAS and replication studies. Vitiligo cases were recruited from clinics of participating dermatologists or self-referred via vitiligo patient societies; for all cases extensive clinical information was available regarding diagnostic criteria, and only cases that met standard diagnostic criteria for generalized vitiligo were included. Non-vitiligo controls were recruited independently. All subjects self-reported age of vitiligo onset. Case self-referral is not likely to affect the results.

Flow Cytometry

Plots

Confirm that:

☒ The axis labels state the marker and fluorochrome used (e.g. CD4-FITC).

☒ The axis scales are clearly visible. Include numbers along axes only for bottom left plot of group (a 'group' is an analysis of identical markers).

☒ All plots are contour plots with outliers or pseudocolor plots.

☒ A numerical value for number of cells or percentage (with statistics) is provided.

Methodology

Sample preparation

To assay leukocyte surface HLA-DR and HLA-DQ proteins, peripheral venous blood was collected into heparinized tubes, and peripheral blood mononuclear cells (PBMC) were isolated by density gradient centrifugation over Ficoll-Paque (GE17-1440-02, Sigma-Aldrich). PBMC were washed and resuspended in RPMI1640 (11875-093, Thermofisher Scientific) containing penicillin-streptomycin (30234250, ThermoFisher Scientific), 40% heat-inactivated FBS (SH30070.03 HI, ThermoFisher Scientific), and 10% DMSO (276855, Sigma-Aldrich) and immediately placed at -80oC in a slow-cooling container. The following day, PBMC were transferred to liquid N2 and were stored until completion of subject recruitment. PBMC aliquots were quick-thawed and resuspended in RPMI1640 (11875-093, ThermoFisher Scientific) with 10% heat-inactivated FBS (SH30070.03 HI, ThermoFisher Scientific) and penicillin-streptomycin (30234250, ThermoFisher Scientific) at a concentration of 10 × 106 cells per mL. 500 µL of PBMC were washed with DPBS (14190-144, ThermoFisher Scientific), incubated with FcR blocking reagent (130-059-901, Miltenyi Biotec) and FACS Staining Buffer—DPBS (14190-144, ThermoFisher Scientific) with 2% BSA (A2153, Sigma-Aldrich) and 10mM EDTA (97062-836, VWR)--then were surfaced-stained with Brilliant Violet 785 anti-CD3 (317330, Biolegend), APC-Cy7 anti-CD14 (367108, Biolegend), Pe-Cy7 anti-CD11b (301322, Biolegend), Brilliant Violet 421 anti-CD11c (301628, Biolegend),

Alexa Fluor 488 anti-CD19 (53-0199-42, eBioscience), PE anti-HLA-DQ (318106, Biolegend), and APC anti-HLA-DR (307610, Biolegend) monoclonal antibodies for 30 min at 4°C. Cells were washed, fixed, resuspended in 2% formaldehyde (28906, ThermoFisher Scientific), and collected using a FACSCelesta flow cytometer and BD FACSDiva software (BD Immunocytometry Systems).

Instrument	FACSCelesta flow cytometer
Software	BD FACSDiva software (BD Immunocytometry Systems)
Cell population abundance	No cell sorting was performed. The average percent abundance of B cells was 5.18%, dendritic cells 5.76%, and monocytes 18.5%.
Gating strategy	Doublets were excluded by gating cells which correlated for FSC-Width and FSC-Area. Debris was excluding by gating on events higher than 25k for FSC and SSC. B cells were identified by bi-modal staining of CD19 and negative for CD3. From the CD3 and CD19 negative events, CD11b and CD14 double positive cells were selected to identify monocytes and CD11b negative but CD11c positive cells to identify dendritic cells. HLA-DR and HLA-DQ expression was monitored by mean fluorescent intensity (MFI) of each immune cell population. When appropriate positive staining and gating strategies were confirmed by fluorescence minus one controls.

☒ Tick this box to confirm that a figure exemplifying the gating strategy is provided in the Supplementary Information.
